# Age‐ and quality‐dependent DNA methylation correlate with melanin‐based coloration in a wild bird

**DOI:** 10.1002/ece3.4132

**Published:** 2018-05-30

**Authors:** Carl D. Soulsbury, Anssi Lipponen, Kristie Wood, Charles A. Mein, Joseph I. Hoffman, Christophe Lebigre

**Affiliations:** ^1^ School of Life Sciences University of Lincoln Brayford Pool Lincoln UK; ^2^ Department of Biological and Environmental Science University of Jyväskylä Finland; ^3^ A. I. Virtanen Institute for Molecular Sciences University of Eastern Finland Kuopio Finland; ^4^ The Genome Centre, Barts and The London School of Medicine and Dentistry Queen Mary University of London London UK; ^5^ Department of Animal Behaviour University of Bielefeld Bielefeld Germany; ^6^ French Research Institute for the Exploitation of the Sea Plouzané France

**Keywords:** *AgRP*, coloration, epigenetics, heterozygosity, melanin, sexual ornament

## Abstract

Secondary sexual trait expression can be influenced by fixed individual factors (such as genetic quality) as well as by dynamic factors (such as age and environmentally induced gene expression) that may be associated with variation in condition or quality. In particular, melanin‐based traits are known to relate to condition and there is a well‐characterized genetic pathway underpinning their expression. However, the mechanisms linking variable trait expression to genetic quality remain unclear. One plausible mechanism is that genetic quality could influence trait expression via differential methylation and differential gene expression. We therefore conducted a pilot study examining DNA methylation at a candidate gene (agouti‐related neuropeptide: *AgRP*) in the black grouse *Lyrurus tetrix*. We specifically tested whether CpG methylation covaries with age and multilocus heterozygosity (a proxy of genetic quality) and from there whether the expression of a melanin‐based ornament (ultraviolet‐blue chroma) correlates with DNA methylation. Consistent with expectations, we found clear evidence for age‐ and heterozygosity‐specific patterns of DNA methylation, with two CpG sites showing the greatest DNA methylation in highly heterozygous males at their peak age of reproduction. Furthermore, DNA methylation at three CpG sites was significantly positively correlated with ultraviolet‐blue chroma. Ours is the first study to our knowledge to document age‐ and quality‐dependent variation in DNA methylation and to show that dynamic sexual trait expression across the lifespan of an organism is associated with patterns of DNA methylation. Although we cannot demonstrate causality, our work provides empirical support for a mechanism that could potentially link key individual factors to variation in sexual trait expression in a wild vertebrate.

## 1 INTRODUCTION

Sexual selection is a key factor driving the evolution of exaggerated sexually selected traits (Darwin, 1871; Andersson, 1994). Sexual traits are often strongly age‐ and condition‐dependent, making them excellent candidates for honest signals that might be used by females to assess male quality; for example, they may carry information about an individual's past and current nutritional status, hormonal status, and/or parasite load (Thompson, Hillgarth, Leu, & McClure, 1997; Ohlsson, Smith, Råberg, & Hasselquist, 2002; Scheuber, Jacot, & Brinkhof, 2003). Furthermore, in many species including birds (Aparicio, Cordero, & Veiga, 2001; Foerster, Delhey, Johnsen, Lifjeld, & Kempenaers, 2003; Ferrer, García‐Navas, Bueno‐Enciso, Sanz, & Ortego, 2015), mammals (von Hardenberg et al., 2007), and fishes (Herdegen, Dudka, & Radwan, 2014), sexual trait expression is associated with genetic quality, as measured by multilocus heterozygosity. Although these associations are as yet poorly understood, a plausible explanation is that they reflect a general tendency for heterozygous individuals to be superior in relation to diverse life history traits (Hansson & Westerberg, 2002) and that heterozygosity influences sexual trait expression indirectly via its effects on body condition.

Another important aspect of sexually selected traits is that their expression tends to be highly variable within individuals and often shows patterns of early life improvement, prime age maximum expression, and senescence (Jones et al., 2008; Nussey et al., 2009; Kervinen, Alatalo, Lebigre, Siitari, & Soulsbury, 2015). Hence, relationships between sexually selected traits and individual quality may vary with age, with the strongest relationships being found during periods of maximal trait expression (Hooper, Tsubaki, & Siva‐Jothy, 1999; Von Hardenberg et al., 2007). Such dynamic patterns of trait expression would not be possible if trait expression was solely under a purely static genetic control; instead epigenetic control mediated by body condition has been proposed as one means of modifying sexual trait expression (Jašarević, Geary, & Rosenfeld, 2012; Valena & Moczek, 2012).

Epigenetics is the study of changes in gene expression and function that cannot be explained by changes in the underlying DNA sequence (Richards, 2006; Bird, 2007). Epigenetic variation can underpin developmental plasticity and canalization which brings about persistent developmental effects in both prokaryotes and eukaryotes (Danchin et al., 2011; Jablonka, 2013). Unlike an individual's genotype, the epigenetic state of an individual is dynamic and can change throughout its lifespan (Horvath, 2013). Such changes can be mediated by environmental variation, exposure to parasites (Wenzel & Piertney, 2014) and hormones (Dhiman, Attwood, Campbell, & Smiraglia, 2015).

The most widespread and stable epigenetic modification is DNA methylation, which refers to the addition of a methyl group (–CH_3_) covalently to the base cytosine (C) in the dinucleotide 5′‐CpG‐3′ (Suzuki & Bird, 2008). Methylation of CpG dinucleotides is generally thought to occlude transcription factor binding, as the methyl groups protrude into the major groove where many transcription factors bind. As a consequence, DNA methylation often acts to silence gene expression (Yin et al., 2017). Furthermore, it is known from human studies that DNA methylation at even single nucleotide positions can alter gene expression dramatically (Pogribny, Pogribna, Christman, & James, 2000).

Epigenetic states are a feature of every organism, and changes in gene expression due to epigenetic effects have the potential to affect numerous important traits (Hill, 2011). There are now a growing number of studies that have examined DNA methylation in wild organisms, mainly in birds (great tits *Parus major*; Riyahi, Sánchez‐Delgado, Calafell, Monk, & Senar, 2015; Derks et al., 2016; Laine et al., 2016; Verhulst et al., 2016; eastern blue birds *Sialia sialis;* Bentz, Sirman, Wada, Navara, & Hood, 2016; red grouse *Lagopus lagopus*; Wenzel & Piertney, 2014; house sparrows *Passer domesitcus*: Liebl, Schrey, Richards, & Martin, 2013; superb starlings *Lamprotornis superbus*; Rubenstein et al., 2016). These and other studies have begun to support a role for DNA methylation in mediating ecological effects on phenotypic traits in the wild (e.g., personality and cognition: Laine et al., 2016; Verhulst et al., 2016) and emphasize the dynamic environmental sensitivity of DNA methylation levels across the life course. However, few if any studies have examined the potential relationship between DNA methylation and sexually‐selected traits, even though epigenetic regulation may represent a critical link between genes and sexually selected trait expression (Jašarević et al., 2012).

In this study, we investigated whether secondary sexual trait expression could be related to patterns of DNA methylation. For this, we exploited a well‐understood genetic pathway—the melanocortin system (Ducrest, Keller, & Roulin, 2008; Roulin & Ducrest, 2013; Roulin, 2016; San‐Jose et al., 2017) in which pigment deposition is directly related to the activity of melanocortin receptors (MCRs). In vertebrates, the principal *MCR* gene expressed in the skin and implicated in melanogenesis is the melanocortin 1 receptor gene (*MC1R*, (Mundy, 2005; Ducrest et al., 2008). Control over expression is achieved via the agonist α*‐melanocortin‐stimulating hormone* (α*‐MSH*) and two inverse agonists *agouti signaling protein* (*AsIP*) and *agouti‐related neuropeptide* (*AgRP*) (Ducrest et al., 2008; Oribe et al., 2012). High expression of *AsIP* and *AgRP* and binding of these inverse agonists induces the production of yellow‐reddish pheomelanic pigments, whereas if the agonist binds to the *MC1R*, then more black eumelanic pigments are produced (Ducrest et al., 2008). Any reduction in the expression of *AgRP* or *AsIP* genes via DNA methylation is thus hypothesized to increase eumelanic (black) coloration.

**Figure 1:**
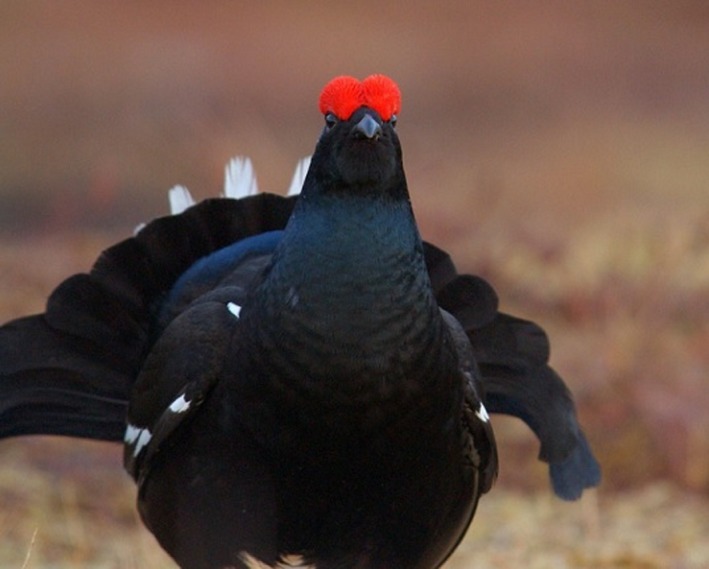
A male black grouse *Lyrurus tetrix* (photo by Gilbert Ludwig)

We tested this hypothesis in a model species for studies of sexual selection in the wild, the black grouse *Lyrurus tetrix* (Figure 1). The dominant coloration of black grouse is eumelanin‐based (black), with the exception of small depigmented patches on the upper and underside of the wing and the conspicuous white undertail coverts (Soulsbury, Kervinen, & Lebigre, 2016). The feathers of the neck and chest also show a blue structural coloration that exhibits high reflectance of short wavelengths in the UV‐blue area (blue chroma; Siitari, Alatalo, Halme, Buchanan, & Kilpimaa, 2007). These sexually selected sexually‐selected traits are condition‐dependent and vary substantially with age (Kervinen et al., 2015; Kervinen, Lebigre, & Soulsbury, 2016). Peak expression occurs at ages 3–5 (Kervinen et al., 2015) and is correlated to male mating success (Siitari et al., 2007; Kervinen et al., 2016). Furthermore, inbreeding is frequent in our study population, with *ca*. 13% of chicks being the product of mating between close relatives (Lebigre, Alatalo, & Siitari, 2010), and both chick mass (Soulsbury, Alatalo, Lebigre, Rokka, & Siitari, 2011) and male reproductive success (Höglund et al. 2001) show inbreeding depression. We therefore estimated the contributions of age and heterozygosity toward DNA methylation and from there tested for a relationship between DNA methylation and sexual trait expression.

## 2 MATERIALS AND METHODS

### 2.1 Fieldwork and color measurement

During 2002–2013 inclusive, we collected longitudinal data on male mating success and multiple sexual traits from five study sites in Central Finland (peat bogs with high visibility, *ca*. 62°15′N; 25°00′E). Data on morphological traits were collected annually in January–March by catching birds from winter flocks with oat‐baited walk‐in traps [for details, see (Kervinen, Alatalo, Lebigre, Siitari, & Soulsbury, 2012; Lebigre, Alatalo, Kilpimaa, Staszewski, & Siitari, 2012)]. Each captured individual was classified either as a yearling or as an adult based on plumage characteristics. Birds were individually ringed for future identification with an aluminum tarsus ring carrying a unique serial number and three colored tarsus rings. All captured birds were blood sampled (<2 ml, maximum <0.3% body mass) with a heparinized syringe from the brachial vein. After centrifugation, the red blood cells were kept in 70% ethanol at 4°C for subsequent DNA analysis. As well as being blood sampled, individuals were measured for body mass, lyre (i.e., tail) length, eye comb size and a representative sample of breast feathers was taken for the measurement of ultraviolet reflectance [blue chroma (Siitari et al., 2007)] using a spectrophotometer.

### 2.2 Microsatellite genotyping and derivation of multilocus heterozygosity

Genomic DNA was extracted from the red blood cells using the reagents from the BioSprint 15 DNA Blood Kit (Qiagen, Ref. 940017) and a Kingfisher magnetic particle processor. All of the individuals were then genotyped at 11 autosomal microsatellite loci (see Lebigre, Alatalo, Siitari, & Parri, 2007 for details). Standardized multilocus heterozygosity (*sMLH*) was calculated based on the 11 autosomal loci (see Soulsbury & Lebigre, 2018) using inbreedR (Stoffel et al., 2016) within R version 3.2.1 (R Core Team 2014). We used *sMLH* as a measure of male genetic quality as heterozygosity is strongly related to both male and female fitness in black grouse (Höglund et al., 2002; Soulsbury & Lebigre, 2018).

### 2.3 Pilot study: characterization of candidate CpG sites

Several genes may be involved in color variation (Nadeau, Burke, & Mundy, 2007; Bourgeois et al., 2016). To focus our study, we therefore initially conducted a pilot study to evaluate the methylation status of selected candidate genes (Table S1). Bisulfite conversion of the DNA was carried out with EZ DNA Methylation‐Gold^™^ Kit D5005 (Zymo Research Corporation, Irvine, CA, USA). Primers for sequencing were designed with MethPrimer (http://urogene.org/methprimer/index.html). PCR products were cleaned with Exo‐SAP (Fermentas), sequenced with a BigDye V3.1 kit (#4336935, Applied Biosystems), and then purified using ethanol precipitation. These were then sequenced on a 3130xl Genetic Analyzer (Applied Biosystems). Using samples from a total of 46 males and females, we tested seven CpG sites across three genes: two CpG sites in the melanocortin‐1 receptor (*Mc1r*), one CpG site in tyrosinase‐related protein 1 gene (*Tyrp1*), and four CpG sites in the agouti‐related protein gene (*AgRP*). The CpG sites within *AgRP* showed the greatest within‐ and between‐individual variation in methylation, whereas the other sites were fully methylated or demethylated, or showed lower variation (Table S1). We therefore focused subsequently on CpG sites within the *AgRP* gene.

### 2.4 Assessment of AGRP methylation

Forward/reverse and sequencing primers for the PCR and pyrosequencing steps, respectively, were designed from modified DNA sequences using the PyroMark Assay Design software version 2.0.1.15 (Qiagen, Uppsala, Sweden). From each sample, 500 ng of genomic DNA was modified with sodium bisulfite (optimal range 200–500 ng) using the EZ DNA Methylation‐Gold^™^ Kit following the manufacturer's instructions (D5005, Zymo Research Corporation, Irvine, CA, USA). PCR was performed on the bisulfite‐converted DNA samples using the forward and reverse primers shown in Table S1. All other reagents were provided as part of the AmpliTaq Gold^®^ DNA Polymerase, LD (low DNA) kit using a Hot Start, Strong Finish^™^ protocol (Applied Biosystems). PCRs were set up in 96‐well plates. All of the samples were processed on a single plate, eliminating the potential for interplate variation. In each well, 4 μl of bisulfite‐converted DNA at 5 ng/μl concentration was added to 50 μl PCR MasterMix containing 2 mM MgCl_2_, and 1 unit of AmpliTaq Gold^®^ DNA polymerase LD (5 units/μl). Plates were processed using a PTC‐225 Peltier Thermal Cycler (MJ Research). Samples were initially incubated at 95°C for 5 min, followed by 45 cycles of 95°C for 15 s, 57°C for 30 s, and 72°C for 5 min. Subsequently, the resultant PCR products were subjected to gel electrophoresis to check that an amplicon of the expected size had been generated.

Pyrosequencing was performed on PCR products using the sequencing primers shown in Table S2. To begin, 20 μl of PCR product from each sample was added to 37 μl Pyromark binding buffer (Ref. 979006, Qiagen) and 3 μl streptavidin sepharose beads (Ref. 17‐5113‐01, GE Healthcare) in a Abgene‐skirted 96‐well plate (Ref. 732‐4888, Merck Ltd, Feltham, Middlesex). A PCR plate seal was applied and the plate was continuously shaken on a high‐speed microplate shaker (Illumina, San Diego, Ca, USA) at 1600 rpm for 20 min. Subsequently, a vacuum Prep workstation (Pyrosequencing®, Qiagen) was used to wash and denature the beads and transfer them to a new plate. Samples were then sequenced on a PSQ 96MA pyrosequencer (Qiagen). PSQ 96MA software version 2.1 (Qiagen) was used to calculate the required amounts of the PyroMark Gold Q96 reagents (Ref. 972804, Qiagen). The substrate mix and enzyme mix were each resuspended in 620 μl MilliQ water prior to being loaded into a PyroMark Q96 cartridge (Ref. 979004, Qiagen) together with the required volumes of dATP, dGTP, dTTP, and dCTP. Pyro‐Q‐CpG software version 1.0.9 (Biotage) was used to analyze the pyrograms in order to determine the percentage of DNA methylation at each individual CpG site by measuring the ratio of the C to T peaks. Control samples were run on each assay. Maximal differences between highest and lowest percent methylation were calculated as follows: site 1 = 6.3%, site 2 = 22.1%, site 3 = 4.0%, site 4 = 4.5%, site 5 = 1.4%.

### 2.5 Statistical analysis

**Figure 2:**
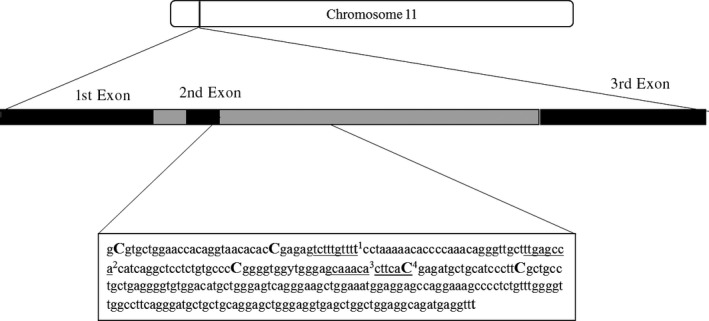
Scaled depiction of the *AgRP* gene showing exons (black bars) and introns (gray bars). Within the sequence, CpG sites are shown in bold and putative transcription binding sites are underlined. Putative transcription binding site names were lifted over from the JASPAR database (http://jaspar.genereg.net/; Mathelier et al., 2016). ^1^Sox3, ^2^ RHOXF1, ^3^FoxD2, ^4^ NKX2‐8

We first tested for differences in the percentage of DNA methylation between sites using a one‐way ANOVA with post hoc Tukey test, followed by correlations between each individual methylation site. We then constructed linear mixed effects models to evaluate the relationships between age, *sMLH,* and the percentage of DNA methylation. As blue chroma exhibits an inverse u‐shaped pattern with age, we fitted age and age^2^ in each of the models, together with their respective interactions with *sMLH*. As only two individuals survived to six years of age, we combined age classes five and six. Each of the CpG sites within the *AGRP* gene was analyzed separately and individual identity was included as a random effect. We analyzed each site singly because, although all of the sites are close together, they differ in respect of whether they are located within introns, exons, or on putative binding sites (Figure 2). Moreover, methylation probabilities at most of the sites were not significantly correlated with each other (Table S3).

Finally, we tested for a relationship between blue chroma and the percentage of DNA methylation in adulthood. We focused only on birds that were at least two years old during the molt after sampling, as sexual traits are not fully expressed in younger males. For this analysis, we analyzed all of the CpG sites within the *AgRP* gene simultaneously within a single model and individual identity was again included as a random effect. All of the models were run using the lme4 package (Bates, Maechler, Bolker, & Walker, 2015) in R version (R Core Team 2014) and none of the models had variance inflation factors (VIF) above two.

## 3 RESULTS

### 3.1 DNA methylation

We sampled 94 males a total of 170 times, at ages varying one to six years old (Table S3). Mean ± SE methylation varied significantly among CpG sites (ANOVA: *F*_4,823_ = 507.26, *p* < .001), with *post hoc* tests showing that sites one, four, and five were significantly different from one another (Site one: 75.94 ± 0.35%; Site four: 72.59 ± 0.32; Site five: 64.90 ± 0.15) while there was no significant difference between the sites two (46.84 ± 1.22%) and three (47.83 ± 0.25%). CpG site methylation was generally uncorrelated among different CpG sites (8/10 correlation coefficients were below 0.30) and the strength of correlation declined with increasing physical distance between dyads of CpG sites (Table S3).

### 3.2 Age‐ and heterozygosity‐dependent patterns of DNA methylation

**Figure 3:**
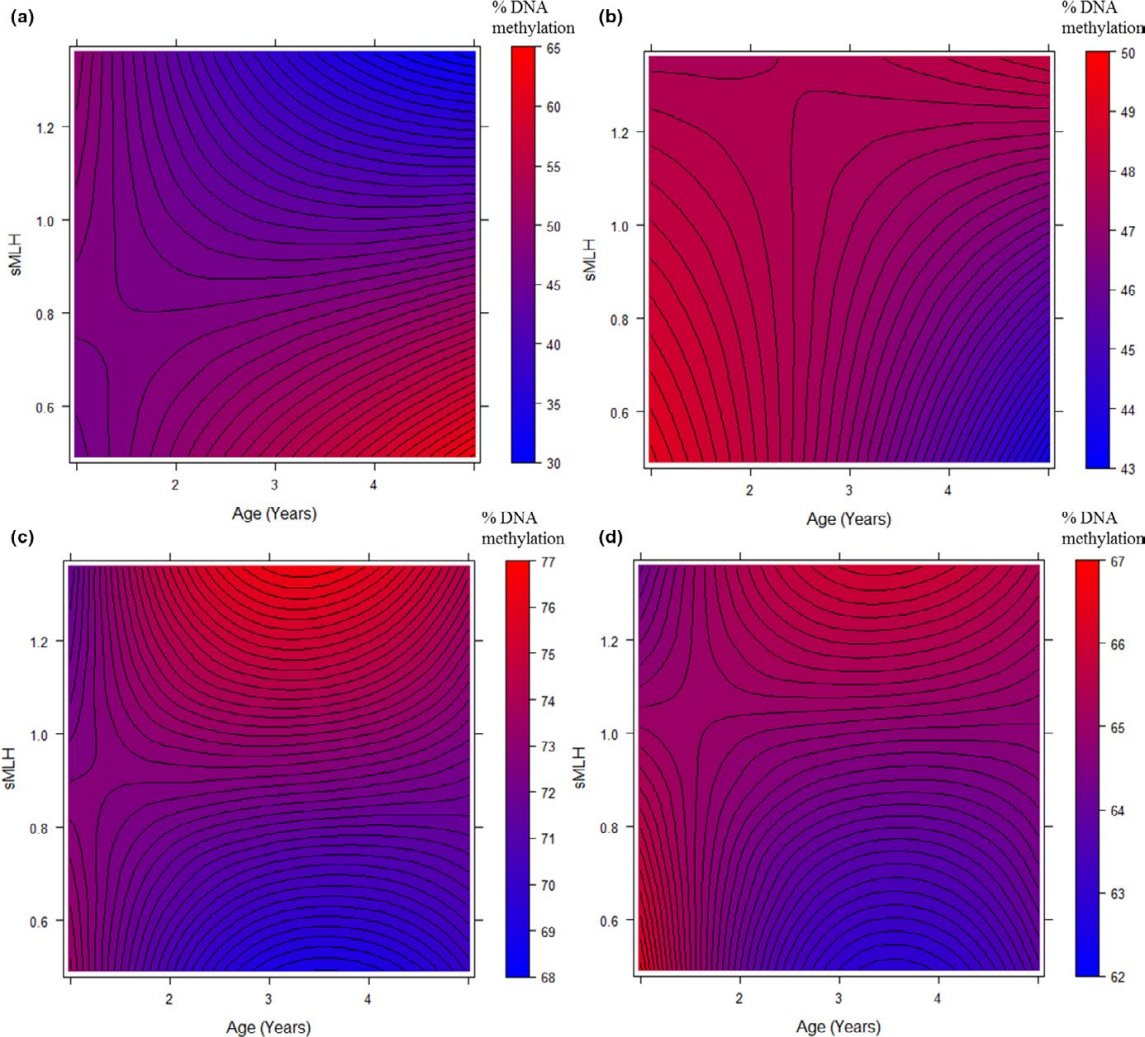
Contour plots showing how DNA methylation varied in relation to age (years) and *sMLH* for CpG sites (a) two, (b) three, (c) four, and (d) five

We next investigated the effects of age and *sMLH* on the percentage of DNA methylation at five different CpG sites within the *AgRP* gene (Table 1, Figure 3). When all of the sites were analyzed together, we found a significant interaction between age and *sMLH* (linear) (Table 1). This pattern was also evident at the level of individual CpG sites. Neither age nor *sMLH* were related to DNA methylation at CpG site one, but a significant interaction between *sMLH* and age was found at CpG site two. More specifically, DNA methylation at this CpG site declined more strongly with increasing *sMLH* when individuals' age increased (*ca*. four years of age or above). For CpG site three, there was a significant effect of age and the age x *sMLH* interaction was close to significance. Contrary to CpG site twp, (contrary to CpG site two, this tendency suggests that DNA methylation increased with *sMLH* in older individuals (Figure 3b). By contrast, CpG sites four and five showed highly consistent patterns with both models retaining significant interactions between *sMLH* and both the linear terms, and either significant (Site 5) or near significant (Site 4) interactions with the quadratic age terms (Table 1). In both cases, there was a positive relationship between methylation and *sMLH* at intermediate ages (three to four years of age, Figure 3c and d).

**Table 1:** Linear mixed effect model outputs for the relationship between DNA methylation at CpG sites within the *AgRP* gene and age, heterozygosity, and their interaction. Age classes five and six were pooled as described in the Methods

CpG site	N males/N samples	Parameter	β	95%CI	*Eff. Sampl*.	*p*
All sites		Age (poly,1)	−36.61	−2.80/−71.37	9000.00	**.035**
		Age (poly,2)	21.14	53.33/−9.61	8144.39	.183
		*sMLH*	0.44	1.82/−0.99	8712.76	.545
		*sMLH* × Age (poly,1)	33.42	67.12/1.56	7687.02	**.042**
		*sMLH* × Age (poly,2)	−21.58	8.40/−53.79	8117.15	0.173
CpG site	N males/N samples	Parameter	β	±SE	*t*	*p*
One	91/161	Age (poly,1)	−9.62	24.17	−0.40	.691
		Age (poly,2)	−13.22	22.48	−0.59	.557
		*sMLH*	−1.11	2.07	−0.54	.595
		*sMLH* × Age (poly,1)	7.46	23.35	0.32	.750
		*sMLH* × Age (poly,2)	16.14	22.04	0.73	.465
Two	91/160	Age (poly,1)	97.68	54.59	1.79	.077
		Age (poly,2)	−50.02	47.21	−1.06	.293
		*sMLH*	−6.67	8.72	−0.77	.446
		*sMLH* × Age (poly,1)	−112.67	52.62	−2.14	**.035**
		*sMLH* × Age (poly,2)	56.60	45.73	1.24	.220
Three	89/157	Age (poly,1)	−38.96	17.02	−2.29	**.024**
		Age (poly,2)	−4.51	15.84	−0.29	.776
		*sMLH*	−0.39	1.56	−0.25	.802
		*sMLH* × Age (poly,1)	31.96	16.46	1.94	.054
		*sMLH* × Age (poly,2)	3.59	15.64	0.23	.819
Four	90/159	Age (poly,1)	−45.49	21.47	−2.12	**.036**
		Age (poly,2)	32.52	20.32	1.60	.112
		*sMLH*	2.11	1.65	1.28	.208
		*sMLH* × Age (poly,1)	48.41	20.71	2.34	**.021**
		*sMLH* × Age (poly,2)	−37.21	19.98	−1.86	.065
Five	83/146	Age (poly,1)	−29.09	9.94	−2.93	**.004**
		Age (poly, 2)	21.92	9.01	2.43	**.016**
		*sMLH*	0.04	0.85	0.04	.967
		*sMLH* × Age (poly,1)	27.73	9.54	2.91	**.004**
		*sMLH* × Age (poly,2)	−21.02	8.97	−2.38	**.019**

### 3.3 DNA methylation and ultraviolet‐blue chroma

**Figure 4:**
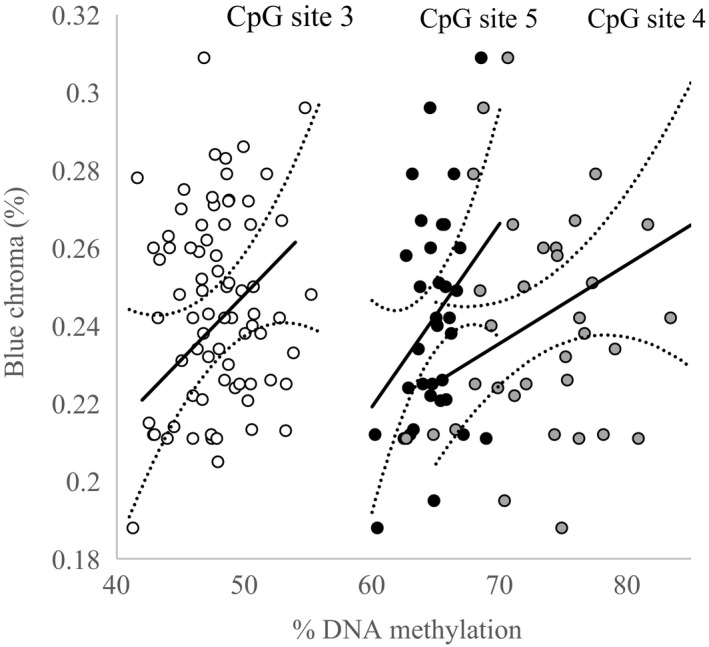
Scatterplot showing the relationship between blue chroma and DNA methylation at CpG sites three, four, and five

The percentage of DNA methylation at CpG sites one and two was unrelated to blue chroma across males aged two or more (CpG site 1: β ± *SE* = 0.001 ± 0.001, *t* = 0.95, *p* = .351; CpG site 2: β ± *SE* = −0.003 ± 0.004, *t* = −0.78, *p* = .447). However, significant positive associations were found between DNA methylation and blue chroma at CpG sites three (β ± *SE* = 0.003 ± 0.002, *t* = 2.14, *p* = .041) and five (β ± *SE* = 0.005 ± 0.002, *t* = 2.16, *p* = .040), with location four showing a similar positive trend that was close to significance (β ± *SE* = 0.002 ± 0.001, *t* = 1.82, *p* = .080; Figure 4).

## 4 DISCUSSION

### 4.1 Aging, heterozygosity, and DNA methylation

Our study reveals clear associations between age, heterozygosity and CpG methylation, which in turn was correlated to sexual trait expression in a natural bird population. In both humans and domesticated animals, there is vast literature characterizing genes or genomic regions that either become hyper‐ or hypomethylated with age (Bollati et al., 2009; Christensen et al., 2009; Bell et al., 2012; Gryzinska, Blaszczak, Strachecka, & Jezewska‐Witkowska, 2013; Gryzinska et al., 2016; Spiers et al., 2016). In wild animals, there is growing interest in age‐specific DNA methylation (Paoli‐Iseppi et al., 2017), but few studies have examined this and the results are often contradictory. For example, DNA methylation shows age‐specific linear changes in humpback whales (*Megaptera novaeangliae*: Polanowski, Robbins, Chandler, & Jarman, 2014) but no changes in superb starlings (Rubenstein et al., 2016), possibly because these studies selected different candidate genes. In black grouse, we found a nonlinear (inverse u‐shaped) pattern of DNA methylation with age, as has similarly been reported in occasional human studies (for an example, see Armstrong, Rakoczy, Rojanathammanee, & Brown‐Borg, 2013). As our study focused primarily on the correlation between DNA methylation and sexual trait expression, such a pattern is to be expected. Sexually selected traits typically show strong inverse u‐shaped responses with age (e.g., Balbontín, De Lope, Hermosell, Mousseau, & Møller, 2011; Kervinen et al., 2015) so factors associated with expression of these traits such as DNA methylation and gene expression might also be expected to show similar patterns.

Our results also suggest a previously unexplored link between DNA methylation and condition dependence, which in the case of black grouse appears to be modulated by multilocus heterozygosity. Several studies have established relationships between elaborate sexual traits and heterozygosity (Foerster et al., 2003; von Hardenberg et al., 2007; Pérez‐González, Carranza, Torres‐Porras, & Fernández‐García, 2010) and these associations tend to be strongest at peak reproductive age (Von Hardenberg et al., 2007). However, our study is the first to our knowledge to have explored the covariation between DNA methylation and sexual trait expression and specifically to attribute age‐ and heterozygosity‐dependent CpG methylation patterns to trait expression. Consequently, even though our study focused on a single candidate gene, it provides intriguing insights that could potentially contribute toward our understanding of the mechanistic basis of sexual trait expression. While gene expression studies are needed to establish causal links between DNA methylation patterns and sexual trait expression, we suspect that our study will be the first of many to uncover such relationships. It is also clear that much more could be learned by extending our approach from a single candidate gene to the entire (epi)genome, which is becoming increasingly feasible thanks to recent advances in the field of genomics.

### 4.2 DNA methylation and melanin coloration

To date, the majority of studies of melanin‐based traits have focused on the genes encoding the melanocortin system and characterized either genetic variation between species (Doucet, Shawkey, Rathburn, Mays, & Montgomerie, 2004; Toews et al., 2016) or how mutations in genes like the *MC1R* are associated with different melanin‐based phenotypes within species (Peters et al., 2016; San‐Jose et al., 2017). So far, only a single study has examined how DNA methylation may impact melanin coloration. This study showed that mice exhibiting a darker (pseudoagouti) phenotype have more methylated CpG sites within a intracisternal A‐particle (IAP) as well as lower levels of *AsIP* expression and darker coloration (Michaud et al., 1994). Our results support this earlier finding because we found no variation among individuals in DNA methylation of the *MC1R* but instead found variation at an antagonist (*AgRP*). *AgRP* may influence melanogenesis in two ways. Firstly, increased CpG methylation at locations within the *AgRP* gene may lead to reduced expression of this inverse agonist and consequently increase blue chroma via increased melanogenesis and consequent changes to the structural coloration (e.g., by increasing the number or density melanin granules, Doucet et al., 2006). Alternatively, *AgRP* could be linked to some other factor, such as body condition or nutritional status (Boswell, Li, & Takeuchi, 2002) that in turn may impact the ability to express the blue chroma.

In avian species with melanin‐based sexual ornaments, there are clear opportunities to further explore the link between DNA methylation and condition dependence. Indeed, studies have shown that melanin colorations are sensitive to sex steroids (Kimball, 2006), that there is pleiotropy between the melanogenic genes and androgens (Ducrest et al., 2008; Béziers, Ducrest, Simon, & Roulin, 2017), and that hormonal stimulation of androgen receptors mediates dynamic changes in DNA methylation patterns at regulatory elements (Dhiman et al., 2015). Furthermore, there are clear links in many species between androgens, immunocompetence and interactions with parasites (Alatalo, Hoglund, Lundberg, Rintamaki, & Silverin, 1996; Mougeot, Irvine, Seivwright, Redpath, & Piertney, 2004; Mougeot, Perez‐Rodriguez, Martinez‐Padilla, Leckie, & Redpath, 2007), suggesting a plausible pathway linking condition via immunity to melanin expression (Gangoso, Roulin, Ducrest, Grande, & Figuerola, 2015). A critical future step will be to carry out integrative studies that characterize all components of this pathway, from condition and its impact on physiology, through DNA methylation and gene expression, ultimately to trait expression.

## 5 CONCLUSION

We examined gene‐specific patterns of DNA methylation in relation to age, genetic quality, and sexual trait expression in a wild animal. Our findings highlight the dynamic nature of DNA methylation and provide insights into age‐ and genotype‐dependent trajectories of sexual trait expression. Although our study is correlative and somewhat preliminary in nature, our findings emphasize that DNA methylation may be a critical component of condition‐dependent sexual trait expression.

## CONFLICT OF INTEREST

None declared.

## AUTHOR CONTRIBUTIONS

CDS and AL conceived the study, CDS and CL carried out field work, CL carried out genotyping, AL carried out pilot epigenetic work. CM and KW carried out pyrosequencing. Statistical analysis was carried out by CDS and CDS wrote the first draft of the MS. CDS, JIH, AL, and CL interpreted the results and wrote the manuscript.

## ANIMAL ETHICS

Birds were captured under the permissions of the Central Finland Environmental Centre (permissions KSU‐2003‐L‐25/254 and KSU‐2002‐L‐4/254) the Animal Care Committee of the University of Jyväskylä (ESLH‐2009‐05181/Ym‐23).

## DATA ACCESSIBILITY

Data available from the Dryad Digital Repository: https://doi.org/10.5061/dryad.7d2m4pp.

## References

[ece34132-bib-0001] Alatalo, R. V. , Hoglund, J. , Lundberg, A. , Rintamaki, P. T. , & Silverin, B. (1996). Testosterone and male mating success on the black grouse leks. Proceedings of the Royal Society B: Biological Sciences, 263, 1697–1702. 10.1098/rspb.1996.0248

[ece34132-bib-0003] Andersson, M. B. (1994). Sexual selection. Princeton, NJ: Princeton University Press.

[ece34132-bib-0004] Aparicio, J. M. , Cordero, P. J. , & Veiga, J. P. (2001). A test of the hypothesis of mate choice based on heterozygosity in the spotless starling. Animal Behaviour, 62, 1001–1006. 10.1006/anbe.2001.1840

[ece34132-bib-0005] Armstrong, V. L. , Rakoczy, S. , Rojanathammanee, L. , & Brown‐Borg, H. M. (2013). Expression of DNA methyltransferases is influenced by growth hormone in the long‐living Ames dwarf mouse in vivo and in vitro. Journals of Gerontology Series A: Biomedical Sciences and Medical Science, 69, 923–933 10.1093/gerona/glt133 PMC4111294 24201695

[ece34132-bib-0006] Balbontín, J. , De Lope, F. , Hermosell, I. G. , Mousseau, T. A. , & Møller, A. P. (2011). Determinants of age‐dependent change in a secondary sexual character. Journal of Evolutionary Biology, 24, 440–448 10.1111/j.1420-9101.2010.02183.x 21175908

[ece34132-bib-0007] Bates, D. , Maechler, M. , Bolker, B. , & Walker, S. (2015). Fitting linear mixed‐effects models using lme4. Journal of Statistical Software, 67, 1–48 10.18637/jss.v067.i01

[ece34132-bib-0008] Bell, J. T. , Tsai, P. C. , Yang, T. P. , Pidsley, R. , Nisbet, J. , Glass, D. , … Shin, S. Y. (2012). Epigenome‐wide scans identify differentially methylated regions for age and age‐related phenotypes in a healthy ageing population. PLoS Genetics, 8, e1002629 10.1371/journal.pgen.1002629 22532803 PMC3330116

[ece34132-bib-0009] Bentz, A. B. , Sirman, A. E. , Wada, H. , Navara, K. J. , & Hood, W. R. (2016). Relationship between maternal environment and DNA methylation patterns of estrogen receptor alpha in wild Eastern bluebird (*Sialia sialis*) nestlings: A pilot study. Ecology and Evolution, 6, 4741–4752 10.1002/ece3.2162 27547309 PMC4979703

[ece34132-bib-0010] Béziers, P. , Ducrest, A.‐L. , Simon, C. , & Roulin, A. (2017). Circulating testosterone and its feather‐gene expression of receptors and metabolic enzymes in relation to melanin‐based coloration in the barn owl. General and Comparative Endocrinology, 250, 36–45 10.1016/j.ygcen.2017.04.015 28457648

[ece34132-bib-0011] Bird, A. (2007). Perceptions of epigenetics. Nature, 447, 396–398 10.1038/nature05913 17522671

[ece34132-bib-0012] Bollati, V. , Schwartz, J. , Wright, R. , Litonjua, A. , Tarantini, L. , Suh, H. , … Baccarelli, A. (2009). Decline in genomic DNA methylation through aging in a cohort of elderly subjects. Mechanisms of Ageing and Development, 130, 234–239 10.1016/j.mad.2008.12.003 19150625 PMC2956267

[ece34132-bib-0013] Boswell, T. , Li, Q. , & Takeuchi, S. (2002). Neurons expressing neuropeptide Y mRNA in the infundibular hypothalamus of Japanese quail are activated by fasting and co‐express agouti‐related protein mRNA.. Molecular Brain Research, 100, 31–42. 12008019 10.1016/s0169-328x(02)00145-6

[ece34132-bib-0014] Bourgeois, Y. X. , Bertrand, J. A. , Delahaie, B. , Cornuault, J. , Duval, T. , Milá, B. , & Thébaud, C. (2016). Candidate gene analysis suggests untapped genetic complexity in melanin‐based pigmentation in birds. Journal of Heredity, 107, 327–335. 26995742 10.1093/jhered/esw017 PMC4888439

[ece34132-bib-0015] Christensen, B. C. , Houseman, E. A. , Marsit, C. J. , Zheng, S. , Wrensch, M. R. , Wiemels, J. L. , … Sugarbaker, D. J. (2009). Aging and environmental exposures alter tissue‐specific DNA methylation dependent upon CpG island context. PLoS Genetics, 5, e1000602 10.1371/journal.pgen.1000602 19680444 PMC2718614

[ece34132-bib-0016] Danchin, É. , Charmantier, A. , Champagne, F. A. , Mesoudi, A. , Pujol, B. , & Blanchet, S. (2011). Beyond DNA: Integrating inclusive inheritance into an extended theory of evolution. Nature Reviews Genetics, 12, 475–486 10.1038/nrg3028 21681209

[ece34132-bib-0017] Darwin, C. (1871). The descent of man and selection in relation to sex. London, UK: John Murray.

[ece34132-bib-0018] Derks, M. F. , Schachtschneider, K. M. , Madsen, O. , Schijlen, E. , Verhoeven, K. J. , & van Oers, K. (2016). Gene and transposable element methylation in great tit (*Parus major*) brain and blood. BMC Genomics, 17, 332 10.1186/s12864-016-2653-y 27146629 PMC4855439

[ece34132-bib-0019] Dhiman, V. K. , Attwood, K. , Campbell, M. J. , & Smiraglia, D. J. (2015). Hormone stimulation of androgen receptor mediates dynamic changes in DNA methylation patterns at regulatory elements. Oncotarget, 6, 42575 10.18632/oncotarget.6471 26646795 PMC4767454

[ece34132-bib-0020] Doucet, S. M. , Shawkey, M. D. , Hill, G. E. , & Montgomerie, R. (2006). Iridescent plumage in satin bowerbirds: Structure, mechanisms and nanostructural predictors of individual variation in colour. Journal of Experimental Biology, 209, 380–390. 10.1242/jeb.01988 16391360

[ece34132-bib-0021] Doucet, S. M. , Shawkey, M. D. , Rathburn, M. K. , Mays, H. L. , & Montgomerie, R. (2004). Concordant evolution of plumage colour, feather microstructure and a melanocortin receptor gene between mainland and island populations of a fairy–wren. Proceedings of the Royal Society B: Biological Sciences, 271, 1663–1670. 15306285 10.1098/rspb.2004.2779 PMC1691780

[ece34132-bib-0022] Ducrest, A.‐L. , Keller, L. , & Roulin, A. (2008). Pleiotropy in the melanocortin system, coloration and behavioural syndromes. Trends in Ecology and Evolution, 23, 502–510. 10.1016/j.tree.2008.06.001 18644658

[ece34132-bib-0023] Ferrer, E. S. , García‐Navas, V. , Bueno‐Enciso, J. , Sanz, J. J. , & Ortego, J. (2015). Multiple sexual ornaments signal heterozygosity in male blue tits. Biological. Journal of the Linnean Society, 115, 362–375. 10.1111/bij.12513

[ece34132-bib-0024] Foerster, K. , Delhey, K. , Johnsen, A. , Lifjeld, J. T. , & Kempenaers, B. (2003). Females increase offspring heterozygosity and fitness through extra‐pair matings. Nature, 425, 714–717. https://doi.org/0.1038/nature01969 14562103 10.1038/nature01969

[ece34132-bib-0025] Gangoso, L. , Roulin, A. , Ducrest, A.‐L. , Grande, J. M. , & Figuerola, J. (2015). Morph‐specific genetic and environmental variation in innate and acquired immune response in a color polymorphic raptor. Oecologia, 178, 1113–1123. 10.1007/s00442-015-3306-6 25834999

[ece34132-bib-0026] Gryzinska, M. , Blaszczak, E. , Strachecka, A. , & Jezewska‐Witkowska, G. (2013). Analysis of age‐related global DNA methylation in chicken. Biochemical Genetics, 51, 554–563. https://doi.org/0.1007/s10528-013-9586-9 23553491 10.1007/s10528-013-9586-9 PMC3712131

[ece34132-bib-0027] Gryzinska, M. , Jakubczak, A. , Listos, P. , Dudko, P. , Abramowicz, K. , & Jezewska‐Witkowska, G. (2016). Association between body weight and age of dogs and global DNA methylation. Medycyna Weterynaryjna, 72, 64–67.

[ece34132-bib-0028] Hansson, B. , & Westerberg, L. (2002). On the correlation between heterozygosity and fitness in natural populations. Molecular Ecology, 11, 2467–2474 10.1046/j.1365-294x.2002.01644.x 12453232

[ece34132-bib-0029] Herdegen, M. , Dudka, K. , & Radwan, J. (2014). Heterozygosity and orange coloration are associated in the guppy (*Poecilia reticulata*). Journal of Evolutionary Biology, 27, 220–225. 10.1111/jeb.12290 24329722

[ece34132-bib-0030] Hill, G. E. (2011). Condition‐dependent traits as signals of the functionality of vital cellular processes. Ecology Letters, 14, 625–634. https://doi.org/0.1111/j.1461-0248.2011.01622.x 21518211 10.1111/j.1461-0248.2011.01622.x

[ece34132-bib-0031] Höglund, J. , Piertney, S. B. , Alatalo, R. V. , Lindell, J. , Lundberg, A. , & Rintamäki, P. T. (2002). Inbreeding depression and male fitness in black grouse. Proceedings of the Royal Society B: Biological Sciences, 269, 711–715. 10.1098/rspb.2001.1937 11934362 PMC1690954

[ece34132-bib-0032] Hooper, R. E. , Tsubaki, Y. , & Siva‐Jothy, M. T. (1999). Expression of a costly, plastic secondary sexual trait is correlated with age and condition in a damselfly with two male morphs. Physiological Entomology, 24, 364–369. 10.1046/j.1365-3032.1999.00152.x

[ece34132-bib-0033] Horvath, S. (2013). DNA methylation age of human tissues and cell types. Genome Biology, 14, 3156 10.1186/gb-2013-14-10-r115 PMC4015143 24138928

[ece34132-bib-0034] Jablonka, E. (2013). Epigenetic inheritance and plasticity: The responsive germline. Progress in Biophysics and Molecular Biology, 111, 99–107. 10.1016/j.pbiomolbio.2012.08.014 22975443

[ece34132-bib-0035] Jašarević, E. , Geary, D. C. , & Rosenfeld, C. S. (2012). Sexually‐selected traits: A fundamental framework for studies on behavioral epigenetics. ILAR Journal, 53, 253–269. 10.1093/ilar.53.3-4.253 23744965 PMC3679548

[ece34132-bib-0036] Jones, O. R. , Gaillard, J. M. , Tuljapurkar, S. , Alho, J. S. , Armitage, K. B. , Becker, P. H. , … Clutton‐Brock, T. (2008). Senescence rates are determined by ranking on the fast‐slow life‐history continuum. Ecology Letters, 11, 664–673. 10.1111/j.1461-0248.2008.01187.x 18445028

[ece34132-bib-0037] Kervinen, M. , Alatalo, R. V. , Lebigre, C. , Siitari, H. , & Soulsbury, C. D. (2012). Determinants of yearling male lekking effort and mating success in black grouse (*Tetrao tetrix*). Behavioral Ecology, 23, 1209–1217. 10.1093/beheco/ars104

[ece34132-bib-0038] Kervinen, M. , Alatalo, R. V. , Lebigre, C. , Siitari, H. , & Soulsbury, C. D. (2015). Life‐history differences in age‐dependent expressions of multiple ornaments and behaviors in a lekking bird. American Naturalist, 185, 13–27. 10.1086/679012 25560550

[ece34132-bib-0039] Kervinen, M. , Lebigre, C. , & Soulsbury, C. D. (2016). Simultaneous age‐dependent and age‐independent sexual selection in the lekking black grouse *(Lyrurus tetrix)* . Journal of Animal Ecology, 85, 715–725. 10.1111/1365-2656.12496 26798985

[ece34132-bib-0040] Kimball, R. T. (2006). Hormonal control of coloration In Hill G. E. & McGraw K. J. (Eds.), Bird coloration. 1. Mechanisms and measurements (pp. 431–468). Cambridge, MA: Harvard University Press.

[ece34132-bib-0041] Laine, V. N. , Gossmann, T. I. , Schachtschneider, K. M. , Garroway, C. J. , Madsen, O. , Verhoeven, K. J. , … Crooijmans, R. P. (2016). Evolutionary signals of selection on cognition from the great tit genome and methylome. Nature Communications, 7, 10474 doi: 10.1038/ncomms10474 PMC4737754 26805030

[ece34132-bib-0043] Lebigre, C. , Alatalo, R. V. , Kilpimaa, J. , Staszewski, V. , & Siitari, H. (2012). Leucocyte counts variation and measures of male fitness in the lekking black grouse. Journal of Ornithology, 153, 95–102 10.1007/s10336-011-0701-6

[ece34132-bib-0044] Lebigre, C. , Alatalo, R. V. , & Siitari, H. (2010). Female‐biased dispersal alone can reduce the occurrence of inbreeding in black grouse (Tetrao tetrix). Molecular Ecology, 19, 1929–1939 10.1111/j.1365-294x.2010.04614.x 20345672

[ece34132-bib-0045] Lebigre, C. , Alatalo, R. V. , Siitari, H. , & Parri, S. (2007). Restrictive mating by females on black grouse leks. Molecular Ecology, 16, 4380–4389 10.1111/j.1365-294x.2007.03502.x 17850264

[ece34132-bib-0046] Liebl, A. L. , Schrey, A. W. , Richards, C. L. , & Martin, L. B. (2013). Patterns of DNA Methylation throughout a range expansion of an introduced songbird. Integrative and Comparative Biology, 53, 351–358. 23535948 10.1093/icb/ict007

[ece34132-bib-0048] Mathelier, A. , Fornes, O. , Arenillas, D. J. , Chen, C. Y. , Denay, G. , Lee, J. , … Zhang, A. W. (2016). JASPAR 2016: A major expansion and update of the open‐access database of transcription factor binding profiles. Nucleic Acid Research, 44, D110–D115 doi: doi.org/10.1093/nar/gkv1176. 10.1093/nar/gkv1176 PMC4702842 26531826

[ece34132-bib-0049] Michaud, E. J. , van Vugt, M. J. , Bultman, S. J. , Sweet, H. O. , Davisson, M. T. , & Woychik, R. P. (1994). Differential expression of a new dominant agouti allele is correlated with methylation state and is influenced by parental lineage. Genes and Development, 8, 1463–1472 10.1101/gad.8.12.1463 7926745

[ece34132-bib-0050] Mougeot, F. , Irvine, J. R. , Seivwright, L. , Redpath, S. M. , & Piertney, S. (2004). Testosterone, immunocompetence, and honest sexual signaling in male red grouse. Behavioral Ecology, 15, 930–937 10.1093/beheco/arh087

[ece34132-bib-0051] Mougeot, F. , Perez‐Rodriguez, L. , Martinez‐Padilla, J. , Leckie, F. , & Redpath, S. M. (2007). Parasites, testosterone and honest carotenoid‐based signalling of health. Functional Ecology, 21, 886–898. 10.1111/j.1365-2435.2007.01302.x

[ece34132-bib-0052] Mundy, N. I. (2005). A window on the genetics of evolution: MC1R and plumage colouration in birds. Proceedings of the Royal Society B: Biological Sciences, 272, 1633–1640. 16087416 10.1098/rspb.2005.3107 PMC1559852

[ece34132-bib-0053] Nadeau, N. J. , Burke, T. , & Mundy, N. I. (2007). Evolution of an avian pigmentation gene correlates with a measure of sexual selection. Proceedings of the Royal Society B: Biological Sciences, 274, 1807–1813. 10.1098/rspb.2007.0174 17504743 PMC2270924

[ece34132-bib-0054] Nussey, D. H. , Kruuk, L. E. B. , Morris, A. , Clements, M. N. , Pemberton, J. M. , & Clutton‐Brock, T. H. (2009). Inter‐ and intrasexual variation in aging patterns across reproductive traits in a wild red deer population. American Naturalist, 174, 42–357. 10.1086/603615 19653847

[ece34132-bib-0055] Ohlsson, T. , Smith, H. G. , Råberg, L. , & Hasselquist, D. (2002). Pheasant sexual ornaments reflect nutritional conditions during early growth. Proceedings of the Royal Society B: Biological Sciences, 269, 21–27. 10.1098/rspb.2001.1848 11788032 PMC1690866

[ece34132-bib-0056] Oribe, E. , Fukao, A. , Yoshihara, C. , Mendori, M. , Rosal, K. G. , Takahashi, S. , & Takeuchi, S. (2012). Conserved distal promoter of the agouti signaling protein (ASIP) gene controls sexual dichromatism in chickens. General and Comparative Endocrinology, 177, 231–237. 10.1016/j.ygcen.2012.04.016 22554923

[ece34132-bib-0057] Paoli‐Iseppi, D. , Deagle, B. E. , McMahon, C. R. , Hindell, M. A. , Dickinson, J. L. , & Jarman, S. N. (2017). Measuring animal age with DNA methylation: From humans to wild animals. Frontiers in Genetics, 8, 106 10.3389/fgene.2017.00106 28878806 PMC5572392

[ece34132-bib-0059] Pérez‐González, J. , Carranza, J. , Torres‐Porras, J. , & Fernández‐García, J. L. (2010). Low heterozygosity at microsatellite markers in Iberian red deer with small antlers. Journal of Heredity, 101, 553–561. 10.1093/jhered/esq049 20478822

[ece34132-bib-0060] Peters, L. , Humble, E. , Kröcker, N. , Fuchs, B. , Forcada, J. , & Hoffman, J. I. (2016). Born blonde: A recessive loss of function mutation in the melanocortin 1 receptor is associated with cream coat colouration in Antarctic fur seals. Ecology and Evolution, 6, 5705–5717. 10.1002/ece3.2290 27547348 PMC4983585

[ece34132-bib-0061] Pogribny, I. P. , Pogribna, M. , Christman, J. K. , & James, S. J. (2000). Single‐site methylation within the p53 promoter region reduces gene expression in a reporter gene construct: Possible in vivo relevance during tumorigenesis. Cancer Research, 60, 588–594. 10676641

[ece34132-bib-0062] Polanowski, A. M. , Robbins, J. , Chandler, D. , & Jarman, S. N. (2014). Epigenetic estimation of age in humpback whales. Molecular Ecology Resource, 14, 976–987 10.1111/1755-0998.12247 PMC4314680 24606053

[ece34132-bib-0063] R Core Team (2014). R: A language and environment for statistical computing. Vienna, Austria: R Foundation for Statistical Computing http://www.R-project.org/.

[ece34132-bib-0064] Richards, E. J. (2006). Inherited epigenetic variation—revisiting soft inheritance. Nature Review Genetics, 7, 395–401 10.1038/nrg1834 16534512

[ece34132-bib-0065] Riyahi, S. , Sánchez‐Delgado, M. , Calafell, F. , Monk, D. , & Senar, J. C. (2015). Combined epigenetic and intraspecific variation of the DRD4 and SERT genes influence novelty seeking behavior in great tit Parus major. Epigenetics, 10, 516–525 10.1080/15592294.2015.1046027 25933062 PMC4622863

[ece34132-bib-0067] Roulin, A. (2016). Condition‐dependence, pleiotropy and the handicap principle of sexual selection in melanin‐based colouration. Biological Reviews, 91, 328–348 10.1111/brv.12171 25631160

[ece34132-bib-0068] Roulin, A. , & Ducrest, A.‐L. (2013). Genetics of colouration in birds. Seminars in Cell & Developmental Biology, 24, 594–608 10.1016/j.semcdb.2013.05.005 23665152

[ece34132-bib-0069] Rubenstein, D. R. , Skolnik, H. , Berrio, A. , Champagne, F. A. , Phelps, S. , & Solomon, J. (2016). Sex‐specific fitness effects of unpredictable early life conditions are associated with DNA methylation in the avian glucocorticoid receptor. Molecular Ecology, 25, 1714–1728 10.1111/mec.13483 26588348

[ece34132-bib-0070] San‐Jose, L. M. , Ducrest, A.‐L. , Ducret, V. , Simon, C. , Richter, H. , Wakamatsu, K. , & Roulin, A. (2017). *MC1R* variants affect the expression of melanocortin and melanogenic genes and the association between melanocortin genes and coloration. Molecular Ecology, 26, 259–276 10.1111/mec.13861 27664794

[ece34132-bib-0071] Scheuber, H. , Jacot, A. , & Brinkhof, M. W. (2003). Condition dependence of a multicomponent sexual signal in the field cricket *Gryllus campestris* . Animal Behaviour, 65, 721–727 10.1006/anbe.2003.2083

[ece34132-bib-0072] Siitari, H. , Alatalo, R. V. , Halme, P. , Buchanan, K. L. , & Kilpimaa, J. (2007). Color signals in the black grouse (*Tetrao tetrix*): Signal properties and their condition dependency. American Naturalist, 169, S81–S92 10.1086/510140 19426093

[ece34132-bib-0073] Soulsbury, C. D. , Alatalo, R. V. , Lebigre, C. , Rokka, K. , & Siitari, H. (2011). Age‐dependent inbreeding risk and offspring fitness costs in female black grouse. Biology Letters, 7, 853–855. 10.1098/rsbl.2011.0379 21632620 PMC3210663

[ece34132-bib-0074] Soulsbury, C. D. , Kervinen, M. , & Lebigre, C. (2016). Curse of the black spot: Spotting negatively correlates with fitness in black grouse *Lyrurus tetrix* . Behavioral Ecology, 27, 1362–1369. 10.1093/beheco/arw057

[ece34132-bib-0075] Soulsbury, C. D. , and Lebigre, C. (2018). Viability selection creates negative heterozygosity–fitness correlations in female Black Grouse Lyrurus tetrix. Journal of Ornithology, 159, 93–101. 10.1007/s10336-017-1474-3

[ece34132-bib-0076] Spiers, H. , Hannon, E. , Wells, S. , Williams, B. , Fernandes, C. , & Mill, J. (2016). Age‐associated changes in DNA methylation across multiple tissues in an inbred mouse model. Mechanisms of Ageing and Development, 154, 20–23. 10.1016/j.mad.2016.02.001 26861500 PMC4798846

[ece34132-bib-0077] Stoffel, M. A. , Esser, M. , Kardos, M. , Humble, E. , Nichols, H. , David, P. , & Hoffman, J. I. (2016). inbreedR: An R package for the analysis of inbreeding based on genetic markers. Methods in Ecology and Evolution, 7, 1331–1339. 10.1111/2041-210X.12588

[ece34132-bib-0078] Suzuki, M. M. , & Bird, A. (2008). DNA methylation landscapes: Provocative insights from epigenomics. Nature Reviews Genetics, 9, 465–476. 10.1038/nrg2341 18463664

[ece34132-bib-0079] Thompson, C. W. , Hillgarth, N. , Leu, M. , & McClure, H. E. (1997). High parasite load in house finches (*Carpodacus mexicanus*) is correlated with reduced expression of a sexually selected trait. American Naturalist, 149, 270–294 10.1086/285990

[ece34132-bib-0080] Toews, D. P. , Taylor, S. A. , Vallender, R. , Brelsford, A. , Butcher, B. G. , Messer, P. W. , & Lovette, I. J. (2016). Plumage genes and little else distinguish the genomes of hybridizing warblers. Current Biology, 26, 2313–2318 10.1098/rspb.2004.2779 27546575

[ece34132-bib-0081] Valena, S. , & Moczek, A. P. (2012). Epigenetic mechanisms underlying developmental plasticity in horned beetles. Genetics Research International, 2012, 14 Article: 576303. 10.1155/2012/576303 PMC3335661 22567393

[ece34132-bib-0082] Verhulst, C. E. , Mateman, A. C. , Zwier, M. V. , Caro, S. P. , Verhoeven, K. J. F. , & van Oers, K. (2016). Evidence from pyrosequencing indicates that natural variation in animal personality is associated with DRD4 DNA methylation. Molecular Ecology, 25, 1801–1811 10.1111/mec.13519 26678756

[ece34132-bib-0083] von Hardenberg, A. , Bassano, B. , Festa‐Bianchet, M. , Luikart, G. , Lanfranchi, P. , & Coltman, D. (2007). Age‐dependent genetic effects on a secondary sexual trait in male Alpine ibex, Capra ibex. Molecular Ecology, 16, 1969–1980 10.1111/j.1365-294x.2006.03221.x 17444905

[ece34132-bib-0084] Wenzel, M. A. , & Piertney, S. B. (2014). Fine‐scale population epigenetic structure in relation to gastrointestinal parasite load in red grouse (Lagopus lagopus scotica). Molecular Ecology, 23, 4256–4273 10.1111/mec.12833 24943398 PMC4282444

[ece34132-bib-0085] Yin, Y. , Morgunova, E. , Jolma, A. , Kaasinen, E. , Sahu, B. , Khund‐Sayeed, S. , … Nitta, K. R. (2017). Impact of cytosine methylation on DNA binding specificities of human transcription factors. Science, 356, eaaj2239 10.1126/science.aaj2239 28473536 PMC8009048

