# Supplementary material for: Age‐ and quality‐dependent DNA methylation correlate with melanin‐based coloration in a wild bird
Source: Ecol Evol. 2018 May 30;8(13):6547–57. doi: 10.1002/ece3.4132 (PMC6053554; doi:10.1002/ece3.4132)
Supplement: Supplementary file 1 [file ECE3-8-6547-s001.docx]

**Supplementary Table S1**: Summary pilot DNA methylation data a 3 genes and 7 CpG sites. Summary data come from 46 adult males and females. All sequences taken from

| **Gene** | **AgRP** | **AgRP** | **AgRP** | **AgRP** | **Mc1r** | **Mc1r** | **Tyr** |
| --- | --- | --- | --- | --- | --- | --- | --- |
| **Ascension number:** | EF571203 EF571163 | EF571203 EF571163 | EF571203 EF571163 | EF571203 EF571163 | EF571037 | EF571037 | EF571128 |
| **CpG site** | CC*GA | CCCC*GGGG | CCCC*A | TTC*G | GGC*(-58) | AACCTT | TTGGC*G |
| **Mean % DNA methylation** | 0.17 | 0.62 | 0.95 | 1 | 1 | 0 | 0.82 |
| **Standard deviation** | ±0.33 | ±0.08 | ±0.14 | ±0 | ±0 | ±0 | ±0.08 |
| **Range** | 0-100% | 50-80% | 50-100% | - | - | - | 65-95% |

| PCR primers | Forward -AGCCAGGAAAGCCCCTCTGTTT | Reverse -GCCAGACTTGGATCAGATGGAGGTGCAT |
| --- | --- | --- |
| Pyrosequencing primers | CAGGCTCCTCTGTGCCC | GAAGGTGATGGTAACCTCCTGCAG |

**Supplementary Table S2**: PCR and pyrosequencing primers

|  | **Site 1** | **Site 2** | **Site 3** | **Site 4** |
| --- | --- | --- | --- | --- |
| **Site 2** | r=-0.42, *P*<0.001 |  |  |  |
| **Site 3** | r=0.20, *P*=0.011 | r=-0.10, *P*=0.224 |  |  |
| **Site 4** | r=-0.22, *P*=0.005 | r=-0.09, *P*=0.256 | r=0.16, *P*=0.048 |  |
| **Site 5** | r=-0.01, *P*=0.936 | r=0.09, *P*=0.271 | r=0.09, *P*=0.265 | r=0.38, *P*<0.001 |

Supplementary Table S3: Pairwise correlation matrix of DNA methylation at each CpG site

Table S4: Samples sizes of males in each age group and the number samples for that age that were also sampled in young age groups.

|  | **1 year old** | **2 years old** | **3 years old** | **4 years old** | **5+years old** |
| --- | --- | --- | --- | --- | --- |
| **Total N males** | 86 | 32 | 25 | 14 | 13 |
| **N males that are sampled in earlier age groups** | - | 27 | 20 | 13 | 5 |
